# Severe hypokalemia induced by ceftazidime-avibactam: Case report

**DOI:** 10.1097/MD.0000000000049288

**Published:** 2026-06-12

**Authors:** Ye Yuan, Yang Gu, Wanjun Bai, Weiqian Liu, Weibo Li, Yu Yin

**Affiliations:** a Department of Rehabilitation, Hebei General Hospital, Shijiazhuang, China; b Hebei Provincial Key Laboratory of Cerebral Networks and Cognitive Disorders, Hebei General Hospital, Shijiazhuang, China; c Department of Pharmacy, Hebei General Hospital, Shijiazhuang, China; d Department of Gastrointestinal Surgery, The Second Hospital of Hebei Medical University, Shijiazhuang, China.

**Keywords:** carbapenem-resistant bacteria, ceftazidime-avibactam, drug-induced hypokalemia, hypokalemia

## Abstract

**Rationale:**

Ceftazidime-avibactam is widely used to treat infections caused by carbapenem-resistant Gram-negative bacteria. Although it is generally considered safe, cases of severe hypokalemia associated with its use have rarely been reported.

**Patient concerns:**

A 74-year-old man with severe pneumonia and acute cerebral infarction was treated with ceftazidime-avibactam after sputum culture and nucleic acid testing identified multidrug-resistant *Acinetobacter baumannii* and *Klebsiella pneumoniae*. Soon after initiation of therapy, he developed persistent and refractory hypokalemia.

**Diagnoses:**

Drug-induced severe hypokalemia secondary to ceftazidime-avibactam was diagnosed.

**Interventions:**

Antimicrobial therapy was continued, and the patient received oral spironolactone and nasogastric potassium chloride. Because of persistent hypokalemia and the development of acute kidney injury, the dose of ceftazidime-avibactam was reduced by half. After dose adjustment and continued potassium supplementation, serum potassium levels gradually stabilized.

**Outcome:**

The patient’s condition worsened due to multiple organ failure and septic shock. At the request of his family, he was discharged against medical advice.

**Lessons:**

This case suggests that avibactam may promote potassium loss by enhancing renal tubular secretion and increasing luminal electronegativity. Clinicians should be aware of this potential adverse effect, particularly in elderly patients or those with renal dysfunction. Close monitoring of serum and urinary potassium levels, along with early correction of abnormalities, is strongly recommended during treatment.

## 1. Introduction

Ceftazidime-avibactam combines a β-lactam antibiotic with a β-lactamase inhibitor and demonstrates potent activity against *Enterobacterales* producing KPC or OXA-48 carbapenemases, as well as multidrug-resistant *Pseudomonas aeruginosa*.^[1]^ Since its approval by the US Food and Drug Administration in 2015 and the European Medicines Agency in 2016, it has become an important therapeutic option for complicated infections caused by carbapenem-resistant Gram-negative bacteria, including hospital-acquired pneumonia and bloodstream infections,^[2]^ particularly in critically ill patients with limited treatment options.

In China, clinical experience has gradually confirmed the efficacy and safety of ceftazidime-avibactam. Studies have reported high rates of both clinical and microbiological response, especially in infections caused by resistant *Klebsiella pneumoniae*.^[3]^ However, as its clinical use increases, concerns about appropriate prescribing have become more prominent. The selection of indications, dosing schedules, and treatment duration still require better standardization.^[4]^

Common adverse reactions include nausea, diarrhea, and mild increases in liver enzymes. Overall, the drug is well tolerated. However, hypokalemia is not recognized as a common adverse reaction in the prescribing information for ceftazidime-avibactam. Recent real-world pharmacovigilance studies have shown that the drug may also be associated with rare but serious adverse events, including cholestasis, drug-induced liver injury, hemolytic anemia, hypernatremia, and severe neutropenia.^[5,6]^ Severe hematologic toxicity, such as pronounced neutropenia, has been reported in case studies.^[7]^ Large pharmacovigilance analyses have also identified other serious reactions as safety signals.^[8]^

Reports describing severe or persistent hypokalemia associated with ceftazidime-avibactam remain extremely limited, and the underlying mechanism is unclear. By contrast, other β-lactam/β-lactamase inhibitor combinations – such as piperacillin/tazobactam, ampicillin/sulbactam, and ceftolozane/tazobactam – have been increasingly associated with hypokalemia, with a reported incidence of up to 40% for piperacillin/tazobactam in some studies.^[9-12]^ This suggests that ceftazidime-avibactam may share similar class-related risks, although direct evidence remains limited. This report describes a patient who developed severe and persistent hypokalemia during treatment with ceftazidime-avibactam for mixed pulmonary infection. Based on the pharmacokinetic characteristics of avibactam, we propose a potential mechanism involving renal tubular ion transport. Specifically, we hypothesize that the negative charge of avibactam may increase the negative potential within the tubular lumen during its active secretion, thereby promoting potassium excretion to maintain electroneutrality. This case provides new insights into the recognition and management of this rare but potentially life-threatening adverse effect in clinical practice and highlights the need for vigilant electrolyte monitoring in elderly patients and those with preexisting renal impairment, in whom tubular compensatory capacity may be diminished.

## 2. Case description

### 2.1. Ethics approval and informed consent

This case report was conducted in accordance with the Declaration of Helsinki. The study was approved by the Ethics Committee of Hebei General Hospital (Approval No. 2024-LW-071). Written informed consent for publication of this case report was obtained from the patient’s family, as the patient was unable to provide consent due to his critical condition. A copy of the consent form is available upon request.

A 74-year-old man was admitted on April 18, 2025, with progressive shortness of breath for 15 days and hematuria for 2 days. He had a history of type 2 diabetes mellitus, hypertension, hyperlipidemia, and prior cerebral infarction. He had been treated in a coronary care unit (CCU) at another hospital on April 5, suggesting recent critical illness and an increased risk of multidrug-resistant (MDR) bacterial colonization. At admission, the diagnoses included severe pneumonia, type I respiratory failure, bloodstream infection with Gram-positive cocci, acute cerebral infarction, coagulopathy, anemia, gastrointestinal bleeding, non-ST-segment elevation myocardial infarction, urinary tract obstruction, and multiple electrolyte imbalances.

**Figure 1.**
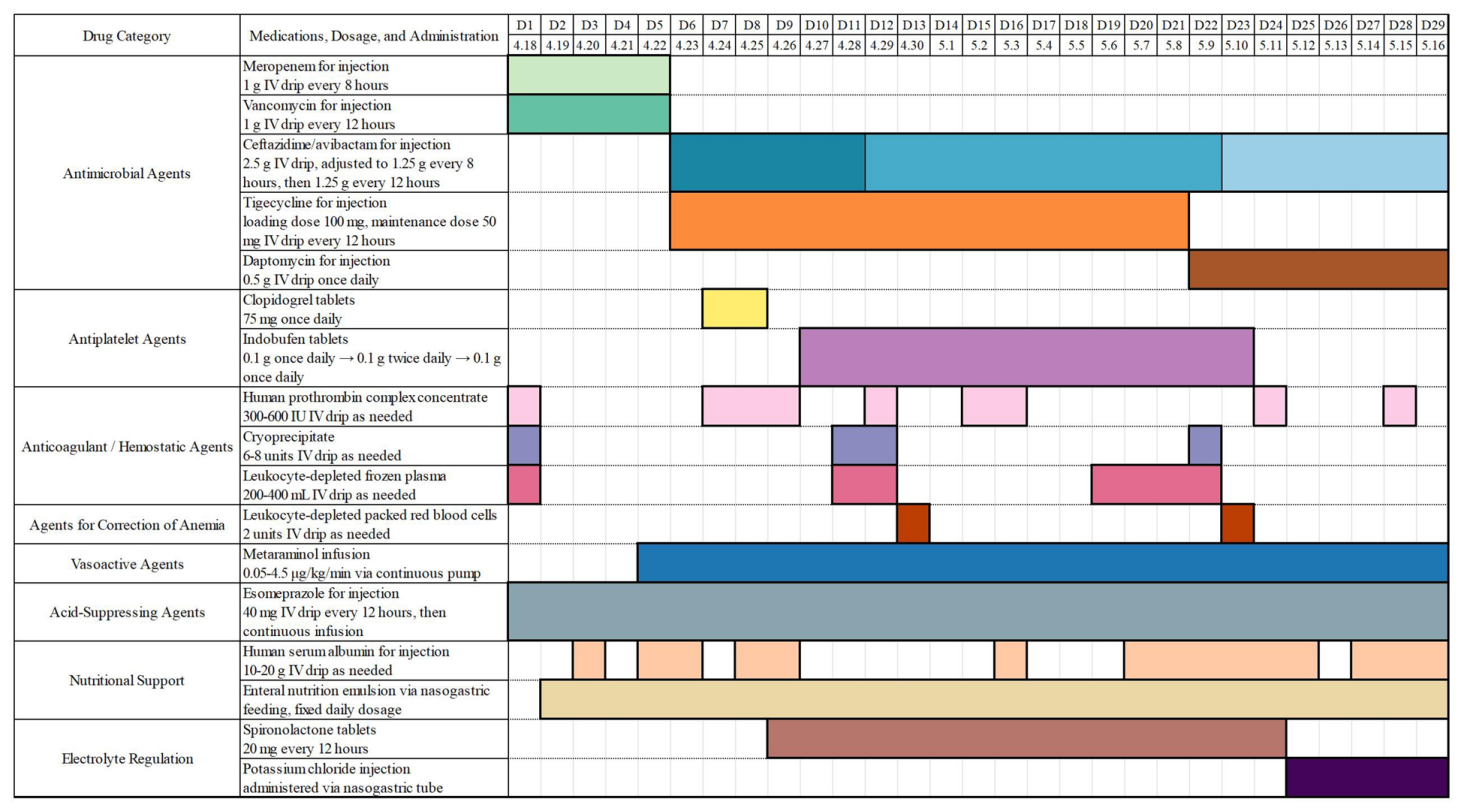
Timeline of the patient’s medication administration during hospitalization. The timeline shows the sequential use of meropenem and vancomycin (April 18–22), followed by ceftazidime-avibactam (2.5 g every 8 hours from April 23) combined with tigecycline. On May 10, the ceftazidime-avibactam dose was reduced to 1.25 g every 12 hours, and tigecycline was gradually discontinued. The onset of hypokalemia (serum potassium < 3.5 mmol/L) began on April 24, the second day of ceftazidime-avibactam therapy. IV = intravenous.

Empirical therapy with meropenem and vancomycin was initiated in view of the patient’s recent CCU stay, severe clinical presentation, and high suspicion of MDR pathogens. On April 22, sputum culture grew *Acinetobacter baumannii* and *K pneumoniae*. Based on susceptibility results and pharmacist consultation, the regimen was changed on April 23 to ceftazidime-avibactam (2.5 g every 8 hours) combined with intravenous tigecycline (Fig. 1). Tigecycline was added to ceftazidime-avibactam to enhance coverage against MDR *A baumannii*, based on susceptibility data and multidisciplinary consultation.

**Figure 2.**
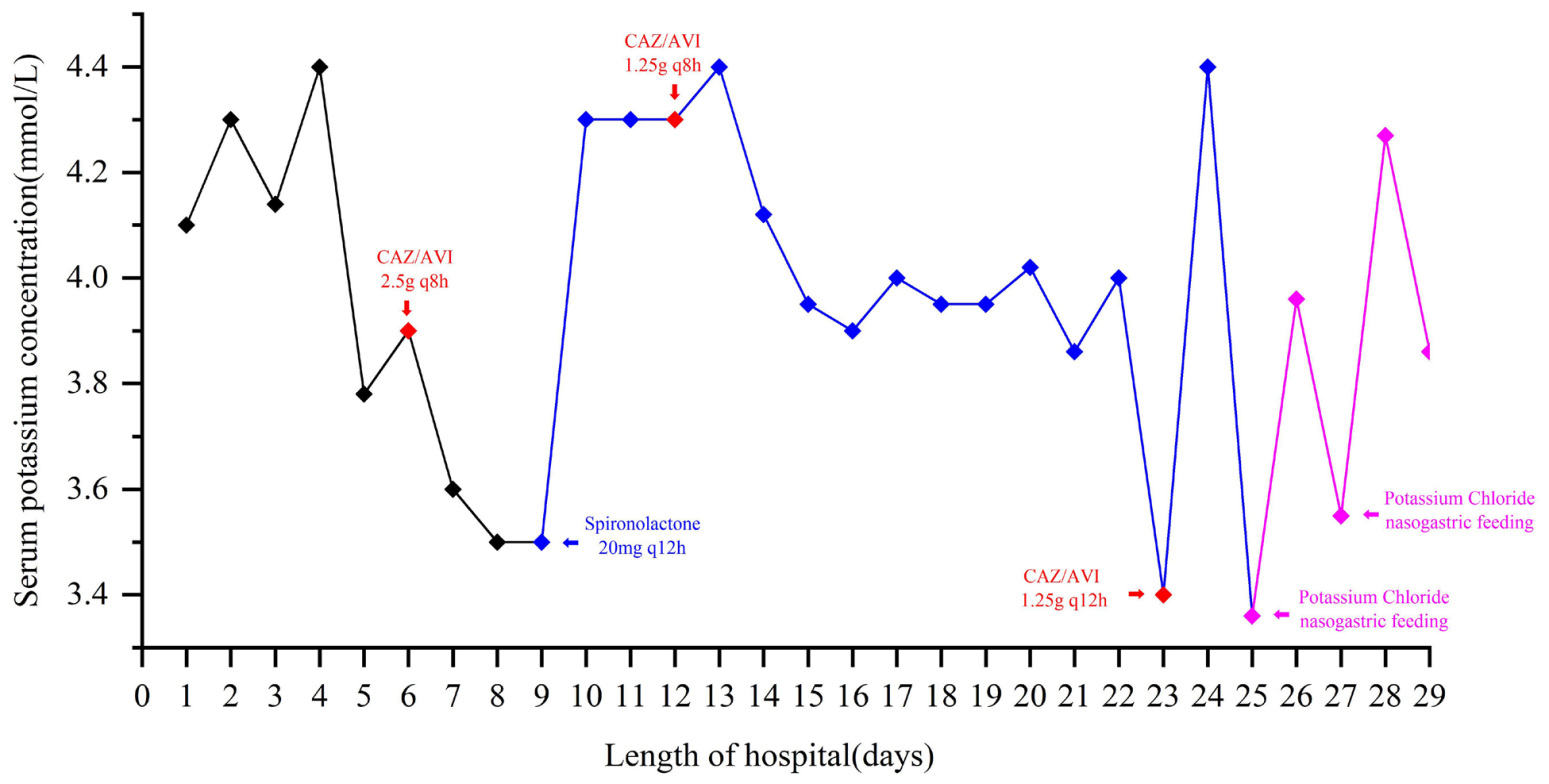
Changes in serum potassium levels before and after ceftazidime-avibactam therapy. Before ceftazidime-avibactam initiation (April 18–22), serum potassium levels remained stable between 4.1 and 4.3 mmol/L. On day 2 of therapy (April 24), potassium decreased to 3.6 mmol/L. Despite oral spironolactone and nasogastric potassium supplementation, potassium further declined to 3.5 mmol/L on April 26 and 3.36 mmol/L by May 12. After dose reduction on May 10, serum potassium gradually stabilized. The dashed line indicates the lower limit of normal (3.5 mmol/L). AVI = avibactam, CAZ = ceftazidime.

Before initiation of ceftazidime-avibactam, serum potassium levels remained within the normal range (4.1–4.3 mmol/L). On the second day of treatment (April 24), potassium decreased to 3.6 mmol/L, indicating mild hypokalemia. As treatment continued, the hypokalemia worsened. On April 26, serum potassium was 3.5 mmol/L, and spironolactone was added to conserve potassium. By May 12, the potassium level fell further to 3.36 mmol/L, with low magnesium and phosphate levels, indicating refractory hypokalemia (Fig. 2).

During this period, there was no evidence of gastrointestinal potassium loss or long-term diuretic use, both of which commonly cause hypokalemia. Notably, the patient had received meropenem for several days before ceftazidime-avibactam, but serum potassium remained stable during meropenem treatment, suggesting that meropenem was unlikely to be the primary contributor to the subsequent hypokalemia. Despite potassium supplementation, the response was suboptimal, and hypokalemia persisted throughout treatment, demonstrating a clear temporal association with ceftazidime-avibactam administration. On May 10, due to worseningrenal function and thrombocytopenia, the ceftazidime-avibactam dose was reduced to 1.25 g every 12 hours, and tigecycline was gradually discontinued on the pharmacist’s advice. After these adjustments, serum potassium levels gradually stabilized, supporting a potential dose-related effect.

## 3. Discussion

This patient developed persistent and refractory hypokalemia temporally associated with ceftazidime-avibactam therapy. According to the Naranjo scale,^[13]^ the total score was 7 (Table 1), indicating a “probable” causal relationship. Although ceftazidime-avibactam is crucial for treating carbapenem-resistant Gram-negative infections, clinicians should closely monitor potential effects on renal electrolyte balance.

**Table 1:** The Naranjo adverse drug reaction assessment scale.

Related questions	Score		
	Yes	No	Unknown
1. Is there any conclusive report before this ADR?	1 √	0	0
2. Do the ADR occur after the use of suspect drugs?	2 √	−1	0
3. Do the ADR relieve after drug withdrawal or use of antagonist?	1	0	0 √
4. Does the ADR recur after reuse of the suspect drug?	2	−1	0 √
5. Are there other reasons that can cause the ADR independently?	−1	2 √	0
6. Does the ADR repeat after the application of placebo?	−1	1	0 √
7. Does the drug reach toxic concentration in blood or other body fluids?	1	0 √	0
8. Is the ADR aggravated (relieved) with the increase (decrease) of dose?	1 √	0	0
9. Has the patient ever been exposed to the same or similar drugs and had similar reactions	1	0	0 √
10. Is there any objective evidence to confirm the reaction?	1 √	0	0
Total score	7		

The scale consists of 10 questions assessing the causality of an adverse drug reaction. Based on the clinical features of this case (temporal relationship, dose–response after reduction, exclusion of alternative causes, and objective evidence), the total score was 7, indicating a “probable” causal relationship between ceftazidime-avibactam and hypokalemia.

The total score ≥ 9 shows that the causal relationship of adverse drug reactions is definite; the total score 5 to 8 is probably or likely to be relevant; the total score 1 to 4 is possible to be relevant; the total score ≤ 0 is doubtful to be relevant.

ADR = adverse drug reactions.

Recent studies have suggested an association between β-lactam antibiotics and hypokalemia. Emerging evidence suggests that β-lactam antibiotics may impair renal tubular function through mechanisms independent of dose, leading to increased urinary potassium excretion.^[9]^ Similar findings have been reported with other β-lactam/β-lactamase inhibitor combinations, including piperacillin/tazobactam^[10]^ and ampicillin/sulbactam.^[11]^ Maraolo et al^[12]^ also found in a systematic review that 4.2% of patients treated with ceftolozane/tazobactam developed hypokalemia. Evidence increasingly suggests that β-lactam/β-lactamase inhibitor combinations may share a class effect on potassium homeostasis, making ceftazidime-avibactam a plausible contributor in this case.

Avibactam is a novel, non-β-lactam β-lactamase inhibitor, and its direct effects on renal tubular function are not yet fully understood. Vishwanathan et al^[14]^ reported that avibactam is primarily excreted by the kidneys. Its renal clearance exceeds the glomerular filtration rate, indicating active tubular secretion that may involve organic anion transporters (OAT1/OAT3). Given its net negative charge, avibactam may increase luminal electronegativity during tubular secretion, thereby promoting potassium excretion to maintain electrochemical balance. This process could increase urinary potassium loss and lower serum potassium levels. This mechanism may be stronger in patients with renal impairment or in older adults, who often have reduced tubular compensation and higher avibactam exposure.

Leegwater et al^[15]^ reported that factors such as female sex, advanced age, concurrent antibiotic use, low body weight, and prolonged therapy were independent risk factors for flucloxacillin-induced hypokalemia. In this patient, advanced age and renal dysfunction likely amplified this effect, as aging kidneys exhibit reduced compensatory capacity for electrolyte regulation.^[16]^

From a mechanistic perspective, Kristensen et al^[17]^ demonstrated in animal models that aldosterone activates epithelial sodium channels (ENaC), which play a central role in potassium loss. In addition, hypokalemia may further stimulate sodium-chloride cotransporter (NCC) expression through feedback mechanisms, forming a vicious cycle. Mutchler et al^[18]^ found that a high sodium intake can modify aldosterone’s regulatory effect on sodium–potassium transport proteins in the distal nephron. These findings suggest that electrolyte imbalance arises from the combined influence of several factors.

Drug-induced tubular dysfunction shares conceptual parallels with inherited tubulopathies such as Bartter and Gitelman syndromes. These parallels support the view that pharmacological interference with tubular ion handling – such as the charge‑mediated effect proposed for avibactam – can produce clinically significant electrolyte disturbances.

To directly link these mechanisms to our patient, we propose the following sequence: avibactam is primarily excreted by the kidneys via active tubular secretion, likely involving organic anion transporters OAT1 and OAT3.^[14]^ At physiological pH, avibactam carries a net negative charge. During its secretion into the tubular lumen, this negative charge may increase the luminal electronegativity. To maintain electroneutrality, the renal tubule enhances potassium excretion – the primary cation available for this purpose. In a young, healthy individual, tubular compensatory mechanisms might partially offset this loss. However, in an elderly patient with reduced renal reserve, as in the present case, this adaptive capacity is impaired, leading to persistent and refractory hypokalemia. This hypothesis is supported by the observation that hypokalemia worsened progressively during treatment and partially stabilized after dose reduction, consistent with a drug concentration‑dependent effect.

Hypokalemia in this patient was clinically meaningful. A serum potassium level below 3.5 mmol/L is widely recognized as clinically significant, and this threshold was crossed repeatedly during ceftazidime-avibactam therapy. The risk of adverse outcomes associated with hypokalemia is well established, particularly in elderly patients with multiple comorbidities.

The temporal relationship in this case further supports causality. Before ceftazidime-avibactam initiation, the patient had received meropenem for several days with stable serum potassium levels (4.1–4.3 mmol/L). Hypokalemia (3.6 mmol/L) emerged on the second day of ceftazidime-avibactam therapy and progressively worsened to 3.36 mmol/L despite oral spironolactone and nasogastric potassium supplementation. Importantly, a dose–response relationship was observed, as hypokalemia improved after dose reduction, further supporting causality. This dose‑response relationship, together with the Naranjo score of 7, further supports a causal role for ceftazidime-avibactam. Although meropenem has been associated with hypokalemia in some reports,^[19,20]^ the stable potassium prior to initiating ceftazidime-avibactam and the partial improvement after dose reduction support ceftazidime-avibactam as the likely contributor.

This phenomenon is unlikely to be unique to patients with cerebral infarction; β-lactam/β-lactamase inhibitor–induced hypokalemia has been documented across different clinical populations, particularly among the elderly or those with compromised renal function.^[12,21]^

Clinically, the patient’s serum potassium stabilized after dose reduction and intensive potassium supplementation with oral spironolactone and nasogastric potassium chloride. This case indicates that even when antimicrobial therapy cannot be stopped, active management can still control electrolyte imbalances effectively. Tai et al^[22]^ described a severe case of hypokalemia in an HIV patient treated with piperacillin/tazobactam and tenofovir alafenamide. The patient’s potassium level returned to normal soon after drug withdrawal, suggesting that drug interactions can worsen potassium loss.

Moreover, drug-induced acute interstitial nephritis (AIN) may also cause severe hypokalemia. Kumar et al^[23]^ reported a case of cephalexin-induced AIN presenting with hypokalemic periodic paralysis. Although AIN was not evident in this case, possible drug-induced tubular injury should still be considered.

## 4. Conclusion

Based on this case and the proposed mechanisms, several practical clinical implications can be drawn. For patients at high risk who require ceftazidime-avibactam therapy, preventive strategies may be considered when appropriate, including early potassium supplementation or the use of potassium-sparing agents such as spironolactone or eplerenone. Close monitoring for early signs of electrolyte imbalance is essential, particularly in elderly patients and those with impaired renal function, as hypokalemia may become refractory if not promptly recognized and managed. However, the effectiveness and safety of these strategies require validation in future prospective studies.

## Acknowledgments

We sincerely thank the patient and his family for their trust and cooperation, and for providing informed consent for publication. We also gratefully acknowledge the Department of Pharmacy, Hebei General Hospital, for their expert clinical assistance and valuable contributions to medication review and data collection. We extend our appreciation to all members of the clinical and laboratory teams whose dedicated efforts were essential to the successful completion of this work.

## Author contributions

**Conceptualization:** Ye Yuan, Yang Gu, Weiqian Liu, Weibo Li.

**Data curation:** Ye Yuan.

**Formal analysis:** Wanjun Bai, Weiqian Liu, Weibo Li.

**Methodology:** Ye Yuan, Wanjun Bai, Weibo Li.

**Writing – original draft:** Ye Yuan, Yang Gu.

**Writing – review & editing:** Yu Yin.
